# Ventral Longitudinal Intraspinal Fluid Collection in Patients with Cervical Disc Herniation: A Report of Two Cases

**DOI:** 10.1155/2020/3439403

**Published:** 2020-03-23

**Authors:** Rui Hirasawa, Takashi Itabashi, Tsuneji Kita, Chikato Mannoji

**Affiliations:** Department of Orthopedic Surgery, Japanese Red Cross Narita Hospital, 90-1, Iidacho, Narita-shi, Chiba 286-8523, Japan

## Abstract

We encountered two cases of cervical disc herniation, wherein cerebrospinal fluid collection in the ventral epidural space between the cervical spine and the thoracic spine was noted. The patients, two women aged 71 and 43 years, were diagnosed with cervical disc herniation and underwent anterior cervical discectomy and fusion. Unexpected cerebrospinal fluid leakage was observed prior to exposure of the dura mater. Notably, the dura mater was intact following the removal of the herniated disc in both cases. No cerebrospinal fluid leakage symptoms were observed, and relief from the neurological symptoms related to the cervical disc herniation was observed in both cases following the surgery. Findings of preoperative magnetic resonance imaging and computed tomography myelography were carefully reviewed, retrospectively. Both patients presented with similar features including expansion of cerebrospinal fluid collection in the ventral epidural space between the cervical spine and the thoracic spine. These observed features were similar to those of superficial siderosis, which is a form of duropathy—a disease caused by dural defects. Therefore, the patients in this case study might have a subclinical duropathy with associated cervical disc herniation.

## 1. Introduction

Epidural arachnoid cysts are one of the most common causes of epidural spinal cerebrospinal fluid (CSF) collection. Epidural arachnoid cysts are primarily located on the dorsal side of the dura and can cause spinal cord compression.

A unique pathological condition, in which CSF collection is observed in the ventral epidural space due to dural defects, has been reported in the recent years [1–5]. Dural hypoplasia, history of trauma, and osteophytes are presumed to be the cause of dural defects and CSF collection. This pathological condition can induce superficial siderosis (SS), craniospinal hypovolemia, or spinal cord herniation and result in clinical symptoms [6]. Kumar defined this pathological condition as duropathy [6].

Herein, we report two cases of cervical disc herniation accompanied by CSF collection in the ventral epidural space, with expansion in the ventral epidural space between the cervical spine and the thoracic spine, which may be associated with duropathy.

## 2. Case Presentation

### 2.1. Case 1

**Figure 1:**
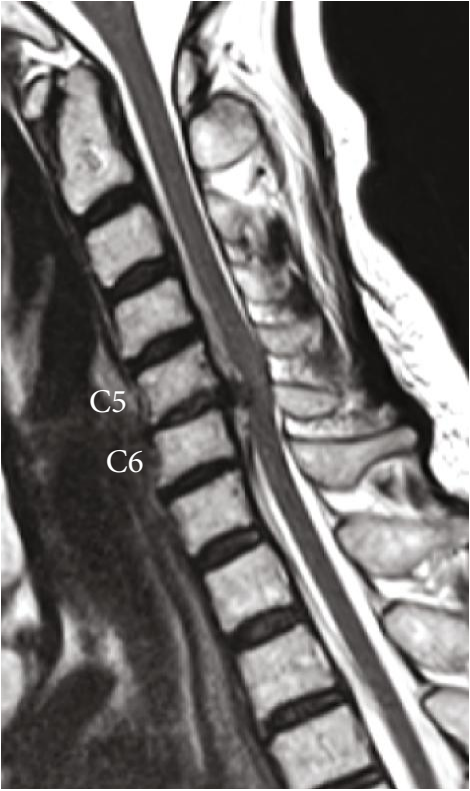
Case 1. Preoperative MRI. T2-weighted sagittal view showing cervical disc herniation at C5-C6 compressing the spinal cord.

A 71-year-old woman was referred to our hospital with a two-week history of cervical pain and numbness in the left upper limb. Neurological examination showed that her left upper limb had a slight motor weakness, with a manual muscle test score of 4. Hoffmann and Wartenberg reflexes were positive in both hands, and deep tendon reflexes in all extremities were hyperactive. However, the patient's left hand displayed reduced dexterity. MRI revealed cervical disc herniation, which compressed the left side of the spinal cord primarily at the C5-6 disc level (Figure 1). Analysis of CSF obtained by a lumbar puncture showed no apparent abnormality and normal protein levels. CSF pressure was not measured. A diagnosis of cervical myelopathy due to cervical disc herniation was established, and an anterior cervical discectomy and fusion (ACDF) at C5-6 was performed.

**Figure 2:**
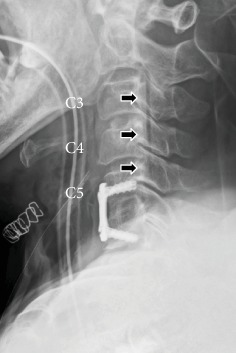
Case 1. Postoperative X-ray (lateral view). A black line (possibly air) was observed posterior to the cervical vertebrae between the bottom of the C2 vertebra and the middle of the C5 vertebra (black arrows).

**Figure 3:**
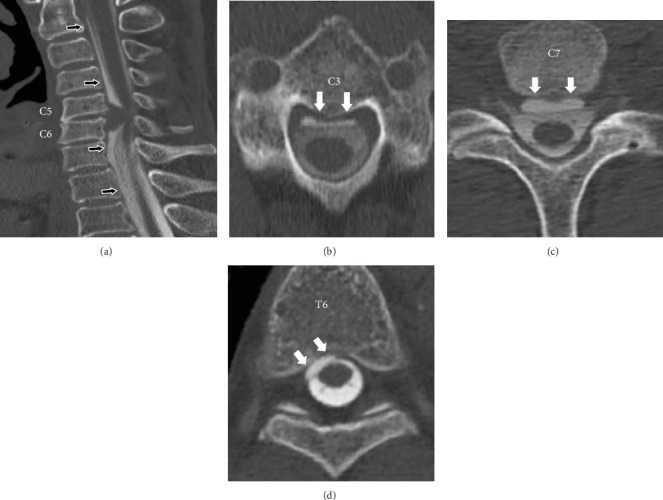
Case 1. Preoperative CT myelography. (a) Sagittal view: fluid collection on the ventral side of the dura (black arrows) adjacent to the cervical disk herniation at C5-C6. (b–d) Axial views: ventral longitudinal intraspinal fluid collection (VLISFC) between C2 and T6 (white arrows).

During the surgery, unexpected CSF leakage was observed when the posterior longitudinal ligament was incised with a scalpel. The dura mater was observed to be intact after the removal of the herniated disc. Thus, it was determined that the membrane that was incised when the CSF leakage occurred was not the dura mater. Fibrin glue was used on the surface of the dura, and no additional treatment of the CSF leakage was performed. Autologous iliac bone was grafted and fixed with a plate and screws. A postoperative X-ray image of the cervical spine revealed a black line posterior to the cervical vertebrae from the bottom of the C2 vertebra to the middle of the C5 vertebra (Figure 2). Preoperative MRI and CT myelography images were carefully reviewed, and CSF collection on the ventral side of the dura expanding from the C2 vertebra to the T6 vertebra was identified (Figure 3). CT myelography is routinely performed 30 minutes after injection of contrast medium. CT myelography was also performed after the surgery and showed that the CSF collection at the surgical site disappeared. However, the CSF collection persisted in the region between levels T2 and T6. An MRI performed 6 months after the surgery showed continued CSF collection between levels C7 and T6. However, no spinal cord compression was observed. Neurological symptoms were relieved after the surgery, and no symptoms related to the CSF leakage were observed. Although the patient was asymptomatic at one year postsurgery, an MRI of the brain was performed, which did not show signs of siderosis, brain sagging, meningeal thickening, or any other abnormality.

### 2.2. Case 2

**Figure 4:**
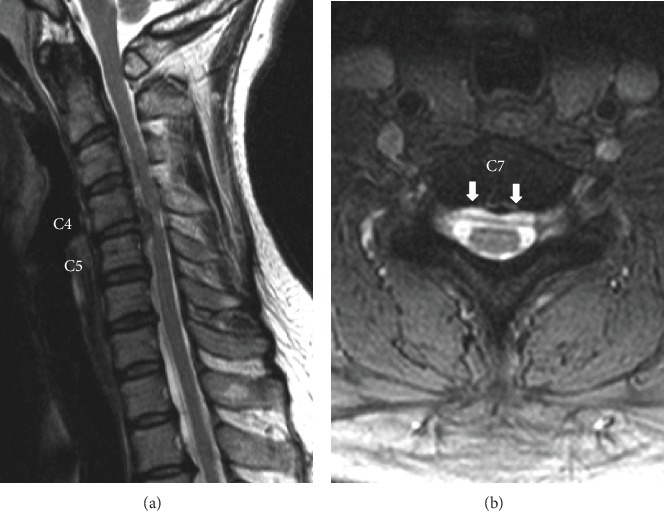
Case 2. Preoperative T2-weighted MRI. (a) Sagittal view: cervical disc herniation compressing the spinal cord was observed at C4-C5. (b) Axial view at the C7 vertebra level: fluid collection was observed on the ventral side of the dura (white arrows).

**Figure 5:**
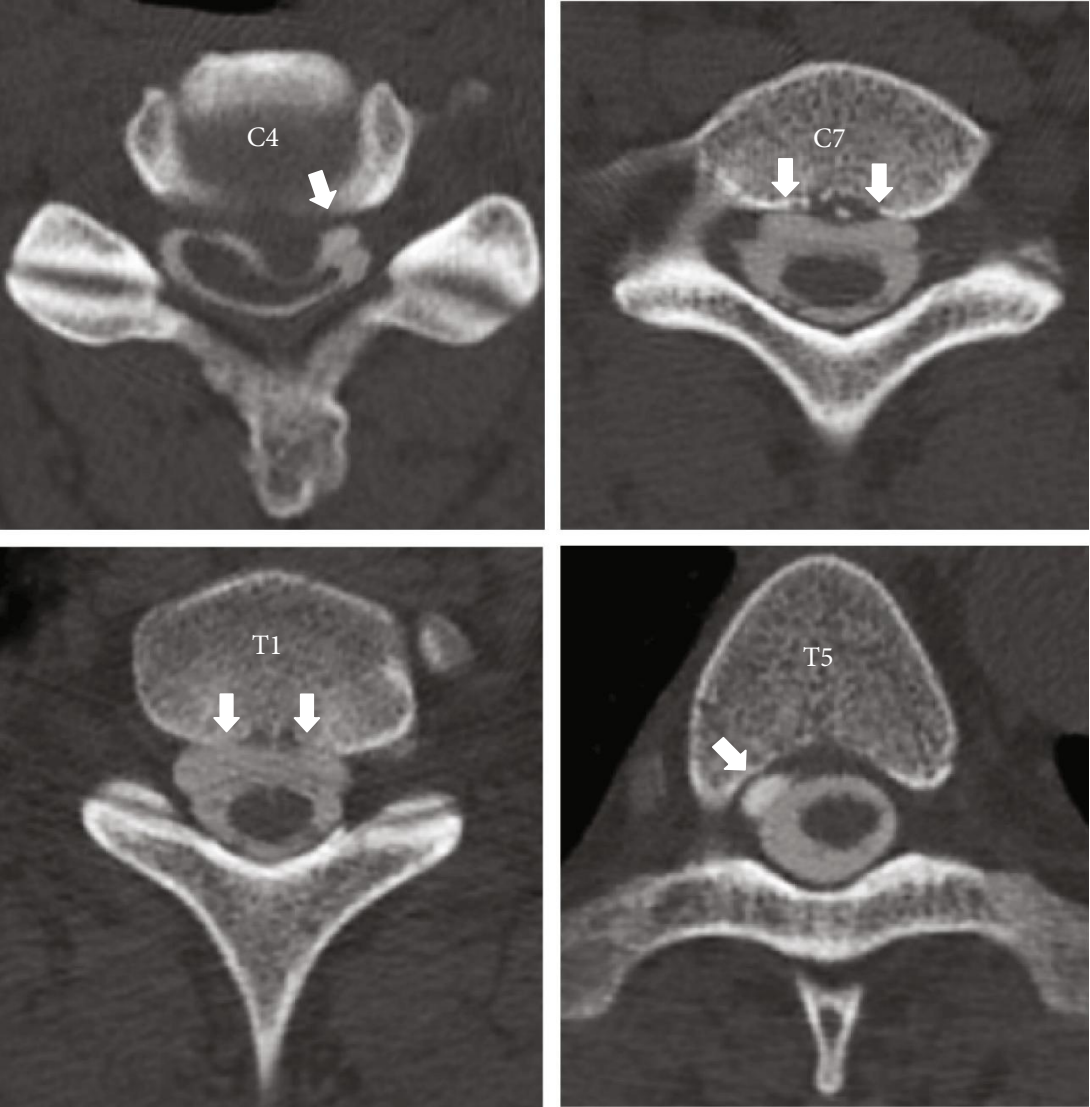
Case 2. Preoperative myelography (axial views): VLISFC (white arrows) between C4 and T5.

A 43-year-old woman was referred to the hospital with a seven-month history of numbness in both upper limbs and the right lower limb and coldness in the right lower limb. While neurological examination showed no muscle weakness, Hoffmann and Wartenberg reflexes were positive in the left hand. Deep tendon reflexes in all extremities were hyperactive. MRI results showed cervical disc herniation, with compression on the left side of the spinal cord, predominantly at the C4-5 disc level (Figure 4(a)). A diagnosis of cervical myelopathy due to the cervical disc herniation at C4-5 was established. Preoperative MRI and CT myelography images showed CSF collection on the ventral side of the dura mater, similar to that seen in Case 1 (Figure 4(b)). CSF collection was observed between levels C4 and T7 (Figure 5). Analysis of CSF obtained by a lumbar puncture showed no apparent abnormality including normal protein levels. CSF pressure was not measured. ACDF was performed at C3-4 and C4-5. C3-4 was included for investigation, as the preoperative X-ray showed instability during flexion and extension of the cervical spine. During surgery, CSF leakage was observed at C4-5 when the posterior longitudinal ligament was incised with a scalpel. Intact dura mater was observed after the removal of the herniated disc. These findings during the surgery were consistent with those described for Case 1. The tissue on the surface of the dura mater was sent for pathological examination. However, only nucleus pulposus tissue was identified. Neurological symptoms were relieved after the surgery, and no further symptoms related to the CSF leakage have been reported.

## 3. Discussions

To our knowledge, there have been no reports of epidural CSF leakage related to cervical disc herniation. However, the visual findings in the cases presented here were similar to cases reported by Kumar et al., which involved CSF collection in the ventral epidural space and were associated with SS [1]. SS is a rare disease that causes hemosiderin deposition in the subpial layers of the brain or spinal cord as a result of chronic or repeated bleeding into the subarachnoid space. Hemosiderin deposition typically causes cerebellar ataxia and sensorineural hearing loss [1, 7]. Kumar et al. reported that CSF collection was observed in 14 of 30 patients with SS. These patients with CSF collection were reported to exhibit dural defects. Additionally, it was observed that the CSF collection was longitudinally extensive and ventrally located in the majority of the patients [1]. Accordingly, this CSF collection was named ventral longitudinal intraspinal fluid collection (VLISFC) [6].

We have reviewed previous reports about VLISFC (Table 1) and found that most VLISFC cases occur between the cervical and thoracic vertebrae. Some reports have attributed VLISFC to dural defects at the same site caused by a protruding disc, osteophytes, a disc-osteophyte complex, or calcification [6, 8]. As a disease associated with dural defects, spinal cord herniation is frequently reported to occur at the thoracic vertebra level, particularly between levels T2 and T8 [9–11]. CSF leakage associated with craniospinal hypovolemia also tends to occur at the thoracic vertebral level and is presumed to be due to dural defects caused by a spondylotic spur or disc herniation [12]. While almost all of the dural defects have been observed at the thoracic spine level in previous reports of VLISFC with associated dural defects (Table 1), there is a possibility that dural defects may also occur at the cervical vertebral level due to osteophytes or disc herniation. However, no dural defects were observed during the anterior cervical surgery in the cases presented herein. With regard to Case 2, protruding osteophytes were observed in CT images at the T2 and T3 vertebral levels. Although the dural defect sites were not identified in the cases presented here, based on previous reports, there is a high possibility that the dural defects existed at the thoracic vertebral level in both cases.

Since the first report by Kumar et al., SS with associated VLISFC has been reported by several institutions [1, 13–15]. In 2012, Kumar et al. also reported cases of VLISFC due to CSF leakage from dural defects in diseases such as SS, craniospinal hypovolemia, and spinal cord herniation [1, 16, 17]. The clinical features of these diseases were reported to overlap and have a common dural lesion [6]. Additionally, a case of SS with associated VLISFC accompanied by spinal cord herniation at the dural defect site was reported by Boncoraglio et al. [9]. Kumar et al. and Holle et al. reported cases of craniospinal hypovolemia related to SS and suggested that craniospinal hypovolemia and SS have similar symptoms, CSF analysis profiles, and imaging findings [18, 19]. Inoue et al. reported a case of spinal cord herniation following craniospinal hypovolemia caused by a dural tear [20]. Kumar has proposed that “duropathy” is a suitable collective term for the group of the diseases caused by these dural lesions [6]. Table 1 shows that VLISFC occur in association with SS, spontaneous intracranial hypotension (SIH) or craniospinal hypovolemia, and spinal cord herniation. Therefore, the pathology of these diseases may overlap due to the presence of similar dural lesions. While previous reports of VLISFC involved symptomatic cases, the two patients in the cases presented here did not experience headaches, cranial nerve symptoms, or cerebellar ataxia, which are sometimes observed in patients with CSF leakage. To our knowledge, the cases presented herein are the first reported cases of asymptomatic VLISFC. However, it is possible that these two patients described are in the stage preceding duropathy and may develop neurological symptoms related to SS, craniospinal hypovolemia, or spinal cord herniation in the future. Therefore, further careful observation is necessary.

There have been reports of patients with VLISFC leading to bibrachial amyotrophy or lower motor neuron weakness. This reported amyotrophy may result from chronic dynamic compression on the spinal cord or nerve conduction block over a tethered motor root segment produced by VLISFC-induced dorsal cord displacement [8]. This proposed mechanism is similar to the pathology of Hirayama disease [21]. In cases presented here, no amyotrophy or lower motor neuron weakness was observed prior to the surgery and preoperative MRI and CT myelography images did not display spinal cord compression due to VLISFC. However, as they still have VLISFC, further careful observation is required.

Although the occurrence of VLISFC may be exceedingly rare, it is notable that it can be identified by MRI or CT myelography. If VLISFC is observed, unexpected CSF leakage may occur during the anterior cervical spine surgery and further careful observation will be required if VLISFC is still observed after surgery.

## 4. Conclusions

In this study, we reported two cases of cervical disc herniation with asymptomatic VLISFC. This pathological condition could represent a precursory stage of duropathy. Hence, SS, craniospinal hypovolemia, or spinal cord herniation may be experienced by the patients in the future. While the occurrence of VLISFC is exceptionally rare, it can be identified by MRI or CT myelography. If VLISFC is observed, unexpected CSF leakage may occur during anterior cervical spine surgery. These findings indicate that further ongoing observation should be provided if VLISFC is still observed after surgery.

## Figures and Tables

**Table 1:** List of published VLISFC cases.

Author (year)	Patient age/sex	Range of VLISFC	Cause of dural defects (level)	Diagnosis
Rabin et al. (1998) [16]	29/female	Entire spine	Not identified	SIH
	27/female	C6-T3	Not identified	SIH
	37/female	C6-T2	Not mentioned	SIH
Kumar et al. (2007) [22]	77/male	Cervical to thoracic spine	Not mentioned	SS and CH
Holle et al. (2008) [18]	59/male	C5-T6	Thoracic disc herniation (T5-6)	SS and CH
Kumar et al. (2009) [19]	64/male	C3-T11	Calcified disc (T7-8)	SS and CH
Payer et al. (2010) [2]	47/female	Cervical to thoracic spine	Calcification (T4)	SS and ALS
Driver-Dunckley et al. (2010) [23]	54/male	C3-L1	Past spinal injury	SS
Schievink et al. (2011) [3]	35/male	C6-T7	Not mentioned	SS and SIH
	22/male	C2-T12	Not mentioned	SIH
	54/female	C6-T10	Not mentioned	SIH
	63/male	T1-T7	Not mentioned	SIH
	37/female	C6-T11	Not mentioned	SIH
	51/male	C3-L5	Not mentioned	SIH
	15/male	C6-T7	Not mentioned	SIH
Cheng et al. (2011) [4]	53/male	C7-T4	Multilobulated arachnoid cyst	SS
Deluca et al. (2011) [8]	48/male	C2-L4	Unknown (T12-L1)	SS
	40/male	C3-L1	Unknown (T11-12)	SS
	32/male	C2-T12	Not mentioned	SS
Boncoraglio et al. (2012) [9]	69/male	C2-T9	Spinal cord herniation (T6-T7)	SS and spinal cord herniation
Kumar et al. (2012) [24]	58/male	Cervical to lumbosacral spine	Calcified disc (T2-3)	SS and CH
Egawa et al. (2013) [13]	67/male	C2-T8	Unknown (T2-3)	SS
	54/male	C7-T8	Unknown (T1-2)	SS
Toro et al. (2014) [5]	66/male	T3-T8	Thoracic disc herniation (T8-9)	SS
Arishima et al. (2017) [14]	50/male	C2-T12	Unknown (T7-8)	SS
	59/male	C2-T12	Unknown (C7)	SS
Takai et al. (2017) [15]	58/male	Cervical to thoracic spine	Unknown (T3)	SS

SIH: spontaneous intracranial hypotension; SS: superficial siderosis; CH: craniospinal hypovolemia; ALS: amyotrophic lateral sclerosis.
